# Household Hints and Recipes

**Published:** 1885-04

**Authors:** 


### Household Hints & Recipes.

INK SPOTS on floors may be removed by rubbing with sand wet in oil of vitrol and water; when the ink is removed rinse with pearl-ash water.

BLACK VARNISH.—Dissolve 50 parts of powdered copal in 400 parts of oil of lavander by the aid of a gentle heat, then add 5 parts of lamp-black and 1 part of powdered indigo.—*Scient. Am.*

TO REMOVE RUST FROM IRON ARTICLES, SUCH AS TACKS, NAILS, &c.—Put the articles into a wire-cloth basket, dip it in dilute sulphuric acid for a very short time, then quickly plunge into boiling water, and next into boiling hot lime water. Finally throw the articles upon a wire-cloth over a fire to dry quickly.—*Scient. Am.*

When lime water, saturated in the cold, is heated, it becomes opalescent, as the lime is much less soluble in hot than in cold water.

GOLD-COLORED SURFACE ON BRASS.—This may be produced with a liquid prepared by boiling together, for about fifteen minutes four parts of caustic soda, four parts of milk-sugar, and one hundred parts of water, to which four parts of a concentrated solution of sulphate of copper should then be added, with constant stirring. The mixture is then cooled to 167° F., and the well-cleansed articles are immersed in it for a short time, when the gold color will appear. A longer immersion results in the formation of a bluish-green tint, and a still more prolonged action causes the formation of iridescent colors.

TO REMOVE TARNISH AND RUST FROM NICKEL-PLATED WARE.—To remove rust from nickel-plated ware without removing the plating, rouge is recommended, which should be applied with chamois skin. A very little oil may be used with the rouge, and the parts may then be wiped with a slightly oily rag. The wiping and polishing must be frequently repeated. Or, take equal parts of precipitated carbonate of iron and prepared chalk, or else take 1 part of mercury with chalk and 4 parts of prepared chalk, and mix them intimately. For use, apply a small quantity with alcohol, and rub with chamois leather.—*Scient. Am.*

REMEDY FOR WARTS.—The following is recommended in the *Journal de Therapeutique:*

Iodine, 12 parts.
Carbolic acid, cryst., 48 parts.
Alcohol, 5 parts.

It is best applied by means of a glass rod.

MENDING MORTARS AND PESTLES.—Melt together equal parts of gutta-percha and shellac in an iron vessel, on the sand-bath; apply a thin coat of the mass upon the strongly heated fractured surface; press forcibly together, and allow to cool, giving the article a suitable position.—*Rundschau* (Leitmeritz.)

TO MEND A WEDGEWOOD MORTAR.—A correspondent of the *Chemist and Druggist* joined the halves of a broken mortar by means of a mixture of calomel and strong mucilage of acacia. The edges were brought closely together, and held in place by means of a string firmly tied just above the rim at the base. The mortar was set aside and not used for some months, after which it was in constant use for a year or two, and appeared to be as strong as ever.

BOTTLE GLUE.—A good bottle glue, insoluble in water, and particularly suitable for sealing bottles containing volatile liquids, such as chloroform, ether, alcohol, etc., may be prepared by soaking glue or gelatin in water, dissolving it in glycerin, then adding tannin (about 2 oz. for every pound of glue) and heating the mixture on a water bath until perfectly homogeneous, and as free from excess of water as possible. It may be colored if desired. When wanted for use it is melted and applied to the mouth of the bottles.—*Trop. Agriculturist.*

FIG PASTE.—Take fifty pounds of white sugar, fifty pounds of crystal, A glucose, and eight gallons of water; put together in a clean kettle and stand it on the fire, and add three ounces of citric acid to it. When it boils mix or stir in fourteen pounds of Duryea's corn starch, dissolved in four gallons of water, keep continually stirring with a wooden spatula on a slow fire for three hours. Try it by putting some of the paste on a cold marble slab to cool; when elastic it is done. Pour in moulds, flavor with lemon for white, rose or strawberry for red; flavor on the fire while boiling. When it is cold cut with a knife in any shape you desire.

To POLISH brass, use ordinary whiting or chalk and a damp cotton or wollen cloth. If the metal is stained or tarnished, then use rottenstone and oil on a cloth, and finish with whiting for a gloss. If corroded and blackened, use oxalic acid in water with the rottenstone, instead of oil.

---

HAIR POMADE.—
Quinine sulphate, 1.
Tannin. 2.
Finest olive-oil, 40.
Cologne, 20.
Cacao butter, 120.
—*Pharm. Zeitsch. f. Russl.*

---

FURNITURE POLISH.—The subjoined simple preparation will be found desirable for cleaning and polishing old furniture:—Over a moderate fire put a perfectly clean vessel. Into this drop two ounces of white or yellow wax. When melted, add four ounces of pure turpentine, then stir until cool, when it is ready for use. The mixture brings out the original color of the wood, adding a lustre equal to that of varnish. By rubbing with a piece of fine cork, it may, when it fades, be removed.

---

FURNITURE-POLISH.—The following is commended as better than the average of such preparations:
Spirit of turpentine, 1 pint.
Rectified oil of amber, 1 pint.
Olive-oil, 1 pint.
Oil of lavender, 1 ounce.
Tincture of alkanet, ½ ounce.
A cotton rubber is saturated with this polish, which is thus applied to the wood. The latter is then well rubbed with soft, dry cotton rags, and wiped dry.

---

MUCILAGE THAT WILL NOT MOULD OR SOUR. —Oil of cloves prevents mouldiness, and sulphate of cinchonine is said to prevent souring.
Gum arabic or dextrine, 8 ounces.
Water, q. s.
Sulphate of cinchonine, 24 grains.
Oil of cloves, 3 drops.
Alcohol, 1 drachm.
Dissolve the gum or dextrine in sufficient water to from a mucilage of the required consistency (about 1 pint.) Mix the sulphate of cinchonine and the oil of cloves with the alcohol, and add to the mucilage.

VARNISH FOR BRIGHT METAL WORK.—Olmsted's varnish, which is a good preventative of rust and oxidation of bright metal work, is made by melting equal parts of rosin and lard together so as to make a soft plastic mass, easily spread on metallic surfaces without lumps. It is best when applied warm.

---

TO FASTEN SHOW-BILLS TO WINDOWS.—Transparent show-bills may be cemented to glass windows in the following manner. Very fine white glue, or preferably clean parchment chippings boiled in distilled water in glass or enamel until dissolved, must be applied very evenly with a soft hair brush to the face of the bill. Then press it on the glass and in a few minutes the bill well be firmly fixed. Glass may be fixed to glass in this way and the cement will bear a good deal of dry heat.

---

EBONIZING WOOD.—Treat repeatedly with strong decoction of logwood or nutgalls. or, better, immerse the wood in the decoction until it has absorbed considerable of the liq[illegible]d. Then paint with a strong solution of copp[illegible]s once or twice, and expose to the sunlight until dry. Then finish with good varnish. The wood should be nicely polished before treatment to insure good results, and it is sometimes necessary to repeat the above operations. This method is given on good authority.

---

CEMENT WHICH RESISTS HEAT AND ACIDS.—This cement consists of a 100 parts of sulphur, 2 parts of tallow, 2 parts of rosin, and a certain quantity of finely powdered and sieved glass. The sulphur, tallow, aud rosin a[illegible]e melted together, until the mass has acquired [illegible] syrupy consistence with a brown color; enoug[illegible] powdered glass is then added, until the ma[illegible] forms a soft dough. The articles to be cemented must first be warmed and the cement must also be applied warm.—*Chem. Zeit.*

---

RUST UPON steel may be removed by covering the rusted part with oil or fat, letting it remain three hours, and wipe off with a cloth: take two drachms caustic potash and fou[illegible] ounces opodeldoc, rub on the mixture, and le[illegible] it remain ten minutes; rub off dry with cloth Or cover the rusted part with sweet oil, wel[illegible] rub in, and next day cover with finely-powdered unslaked lime, and polish with this unt[illegible] the rust disappears. Or take half an ounce o[illegible] emery powder and one ounce of soft soa[illegible] mixed, and rub well.

